# Multiple impacts of the COVID-19 pandemic and antimicrobial stewardship on antimicrobial resistance in nosocomial infections: an interrupted time series analysis

**DOI:** 10.3389/fpubh.2024.1419344

**Published:** 2024-07-17

**Authors:** Weibin Li, Xinyi Yang, Chaojie Liu, Xu Liu, Lin Shi, Yingchao Zeng, Haohai Xia, Jia Li, Manzhi Zhao, Shifang Yang, Xiaojie Li, Bo Hu, Lianping Yang

**Affiliations:** ^1^School of Public Health, Sun Yat-sen University, Guangzhou, China; ^2^School of Psychology and Public Health, La Trobe University, Melbourne, VIC, Australia; ^3^Department of Infectious Disease, The Fifth Affiliated Hospital, Sun Yat-sen University, Zhuhai, China; ^4^Department of Pharmacy, The First Affiliated Hospital, Sun Yat-sen University, Guangzhou, China; ^5^Department of Pulmonary and Critical Care Medicine, Guangdong Provincial People's Hospital, Guangdong Academy of Medical Sciences, Southern Medical University, Guangzhou, China; ^6^Department of Laboratory Medicine, The Third Affiliated Hospital, Sun Yat-sen University, Guangzhou, China

**Keywords:** antimicrobial resistance, COVID-19, interrupted time series, China, antimicrobial stewardship

## Abstract

**Objectives:**

The emergency response to the COVID-19 pandemic may disrupt hospital management activities of antimicrobial resistance (AMR). This study aimed to determine the changing AMR trend over the period in China when stringent COVID-19 response measures were implemented.

**Methods:**

This retrospective study was conducted in a designated hospital for COVID-19 patients in Guangzhou, China from April 2018 to September 2021. The prevalence of 13 antimicrobial-resistant bacteria was compared before and after the COVID-19 responses through Chi-square tests. Interrupted time series (ITS) models on the weekly prevalence of AMR were established to determine the changing trend. Controlled ITS models were performed to compare the differences between subgroups.

**Results:**

A total of 10,134 isolates over 1,265 days were collected. And antimicrobial-resistant strains presented in 38.6% of the testing isolates. The weekly AMR prevalence decreased by 0.29 percentage point (95% CI [0.05–0.80]) after antimicrobial stewardship (AMS) policy, despite an increase in the prevalence of penicillin-resistant *Streptococcus pneumoniae* (from 0/43 to 15/43, *p* < 0.001), carbapenem-resistant *Escherichia coli* (from 20/1254 to 41/1184, *p* = 0.005), and carbapenem-resistant *Klebsiella pneumoniae* (from 93/889 to 114/828, *p* = 0.042). And the changing trend did not vary by gender (male vs. female), age (<65 vs. ≥65 years), service setting (outpatient vs. inpatient), care unit (ICU vs. non-ICU), the primary site of infection (Lung vs. others), and Gram type of bacteria (positive vs. negative).

**Conclusion:**

The response to COVID-19 did not lead to an increase in overall AMR; however, it appears that management strategy on the prudent use of antimicrobials likely contributed to a sizable long-term drop. The frequency of several multidrug-resistant bacteria continues to increase after the COVID-19 epidemic. It is crucial to continue to monitor AMR when COVID-19 cases have surged in China after the relaxation of restriction measures.

## Introduction

Antimicrobial resistance (AMR) is regarded as one of the most pressing public health concerns. Evidence suggests that inappropriate use of antibiotics is associated with rising resistance, which has selectively pressured microorganisms to develop resistance mechanisms (1, 2). The resistant strains can no longer be controlled with standard treatments, leading to prolonged illnesses, increased mortality rates, and higher healthcare expenditures (3). The extended duration of illness and increased severity of symptoms associated with AMR infections necessitate more intensive and resource-intensive medical interventions, such as additional hospital admissions, surgical procedures, and supportive care (1). Furthermore, the loss of effective first-line treatments means that health systems must invest in new drugs and technologies, which can be economically burdensome (4).

An estimated 4.95 million deaths were attributable to AMR globally in 2019 (3). The COVID-19 pandemic has introduced a new layer of complexity to the challenges posed by AMR (5). The increased use of antibiotics to manage COVID-19 complications, such as secondary bacterial infections, has the potential to exacerbate existing trends in AMR (6). Furthermore, the pandemic has strained healthcare resources, disrupted supply chains for essential medications, and diverted attention and funding away from AMR initiatives (7). This has made it even more difficult for medical workers to adhere to antibiotic prescription guidelines and implement effective infection control measures (8).

China is one of the countries in the world that introduced the strictest restriction measures to fight against COVID-19 (9). In China, the “dynamic zero-COVID” policy has necessitated rigorous infection prevention and control measures, which, while crucial for containing the spread of the virus, have also presented challenges for the management of AMR (10). The need for frequent nucleic acid testing and the protocol for treating patients with fever have complicated the submission of clinical samples for microbiological testing and the enforcement of antibiotic stewardship programs. Evidence from China and other regions has highlighted the impact of the COVID-19 pandemic on hospital-acquired infections (HAIs) and antibiotic consumption patterns (11–13).

There was much empirical evidence of influenced hospital-acquired infections (HAIs) and antibiotic consumption during the COVID-19 pandemic in China. For instance, a higher HAI rate was reported in Nanjing, China in 2020 (14). A similar study found that during the COVID-19 epidemic, antibiotic use increased by 137% and antibiotic use patterns significantly changed among a rural community in Eastern China (15). However, the analysis of nationwide procurement data revealed a different result that antibiotic-defined daily doses per 1,000 inhabitants per day dropped in 2020 (16).

However, the studies focused on AMR are relatively limited and mostly among COVID-19 patients. A systematic review included 148 studies that revealed a proportion resistant to antimicrobials 60.8% among COVID-19 patients with bacterial infection people with bacterial infections, the overall changes of AMR are still unknown because of the limited evidence among non-COVID-19 patients. Additionally, the majority of cross-sectional studies make it simple to get contradicting conclusions based on different periods. In fact, after the COVID-19 outbreak, the National Health Commission of China issued a series of regulations and notices in 2020, attempting to strengthen hospital infection control and rational use of antimicrobials (17–19). As a result, it is necessary to assess the dynamic changes of AMR through interrupted time series design, which is considered one of the strongest quasi-experimental designs (20). Our study aimed to determine the changing trend of AMR over the period in China when stringent COVID-19 response measures were put in place. The findings of our study will also offer some baseline information for future research into the AMR trend under the new context of COVID-19 in China (21).

## Methods

### Design and setting

The study adopted an interrupted time series design and was conducted in a large tertiary hospital affiliated with Sun Yat-sen University in Guangzhou, China. The hospital has 2000 beds and receives more than 14,000 outpatient and emergency visits per day on average. The hospital was arranged as one of the COVID-19 designated hospitals after the disease was first identified in Guangzhou on 21st January 2020, and has taken strict triage and isolation measures under the guidance of the Guangdong Provincial Health Commission (22). During the initial COVID-19 outbreak, due to the response to the pandemic such as isolation, adjustment of patient treatment guidelines, and reallocation of hospital resources, the number of patients in the hospital decreased, resulting in a decrease in the number of specimens submitted, but daily service has been resumed in the most of departments since late February 2020 (23).

### Data collection

AMR testing data were extracted from the microbial laboratory information system and linked back to the hospital infection control system that recorded the source of the testing samples. We picked the samples of regular bacteria monitored by the China Antimicrobial Resistance Surveillance System (CARSS) after eliminating duplicate samples from the same patient. Finally, a total of 10,134 isolates over 1,265 days (from April 2018 to September 2021) were collected, covering nine strains of bacteria: *Staphylococcus aureus* (*S. aureus*), coagulase-negative *staphylococcus*, *Enterococcus faecalis* (*E. faecalis*), *Enterococcus faecium* (*E. faecium*), *Streptococcus pneumoniae* (*S. pneumoniae*), *Escherichia coli* (*E. coil*), *Klebsiella pneumoniae* (*K. pneumoniae*), *Pseudomonas aeruginosa* (*P. aeruginosa*), and *Acinetobacter baumannii* (*A. baumannii*).

The following 13 types of AMR were tested: methicillin resistant *S. aureus* (MRSA), methicillin resistant coagulase-negative staphylococci (MRCNS), vancomycin resistant *E. faecalis* (VR-efa), vancomycin resistant *E. faecium* (VR-efm), penicillin resistant *S. pneumoniae* (PRSP), erythromycin resistant *S. pneumoniae* (ERSP), third generation cephalosporin resistant *E. coli* (CtxCroR-eco), carbapenem resistant *E. coli* (CR-eco), quinolone resistant *E. coli* (QREC), third generation cephalosporin resistant *K. pneumoniae* (CtxCroR-kpn), carbapenem resistant *K. pneumoniae* (CR-kpn), carbapenem resistant *P. aeruginosa* (CR-pae) and carbapenem resistant *A. baumannii* (CR-aba). The susceptibility breakpoints followed the Clinical and Laboratory Standards Institute (CLSI) 2019 guidelines (24).

### Statistical analysis

The prevalence of AMR was calculated and compared before and after the launch of COVID-19 responses using Chi-square tests. Interrupted time series analyses with a Poisson segmented regression model were performed to determine both step and trend changes in the weekly prevalence of AMR. Two intervention (interruption) points were set up in the analyses: one on January 23, 2020, when Guangdong Province launched the top-level response to a major public health emergency, and another on July 23, 2020, when the series of national regulations and notices relevant to AMR was first released (Table 1).

**Table 1:** Regulations and notices issued by the National Health Commission in response to the COVID-19 outbreak.

Date	Regulations and notices	Key points
2020-03-13	Further strengthening the prevention and control of infections in medical institutions during the epidemic period (IPC* policy)	Hand hygiene Active surveillance of infections Infection isolation
2020-06-30	Improving infection prevention and control in medical institutions and fever clinics (Second IPC policy)	Adding fever clinics Strengthening infection training Allocation of professional physicians of infectious diseases
2020-07-23	Continuous management of clinical use of antimicrobial agents (AMS* policy)	Special management of key antimicrobial agents Standardizing the collection and transportation of microbial samples

*IPC, infection prevention and control practices; AMS, antimicrobial stewardship.

Potential confounding factors, including patient and sample characteristics and seasonal factors [spring: c(0,0,0), summer: c(1,0,0), autumn: c(0,1,0), winter: c(0,0,1)], were introduced into the models as covariates. Considering the effect of the number of patients on resistance, we added the number of specimens submitted as an offset to the model to balance its effect on resistance rates. The evaluated parameters in the model contained baseline trend (trend before the intervention), step change (short-term change of the intervention), and trend change (long-term change in trend after intervention). The effect estimates were expressed as a weekly percentage change (WPC) and incidence rate ratio (IRR and 95% confidence interval).

We also conducted subgroup analyses by dividing the study sample by gender (male vs. female), age (<65 vs. ≥65 years), service setting (outpatient vs. inpatient), care unit (ICU vs. non-ICU) of the patients, the primary site of infection (lung vs. others), and the Gram type of bacteria (positive vs. negative).

All statistical analyses were conducted using R software (version 4.1.3). A two-sided *p*-value of less than 0.05 was considered statistically significant.

## Results

Of the 10,134 isolates, 3,912 (38.6%) were identified with AMR, close to the national average prevalence level reported by CARSS in 2020. Although more AMR isolates were identified before (*n* = 2,057) than after (*n* = 1,855) the launch of COVID-19 measures (see details of the sample distribution of the AMR isolates in Supplementary Table S2), Chi-square tests detected no significant changes in the prevalence of AMR before and after the launch of COVID-19 measures (*p* = 0.366), except for an increase of the prevalence of PRSP (increased from 0 to 34.9%, *p* < 0.001), CR-eco (increased from 1.6 to 3.5%, *p* = 0.005) and CR-kpn (increased from 10.5 to 13.8%, *p* = 0.042) within the study period (Table 2).

**Table 2:** Prevalence of antimicrobial resistance before and after the launch of COVID-19 measures.

Bacterial strain	CARSS^*^ 2020 (%)	Number and percentage (%) of resistant isolates			
		Total	Pre-COVID	Post-COVID	*p*
**Gram-positive**					
MRSA	29.4	422 (37.0)	212 (36.0)	210 (38.0)	0.512
MRCNS	74.7	922 (72.5)	500 (73.4)	422 (71.0)	0.459
VR-efa	0.2	2 (0.4)	1 (0.4)	1 (0.4)	1.000
VR-efm	1.0	6 (1.4)	3 (1.3)	3 (1.4)	1.000
PRSP	0.9	15 (17.4)	0 (0)	15 (34.9)	<0.001
ERSP	96.0	73 (84.9)	39 (90.7)	34 (79.1)	0.229
**Gram-negative**					
CtxCroR-eco	51.6	1,379 (56.6)	700 (55.8)	679 (57.4)	0.472
CR-eco	1.6	61 (2.5)	20 (1.6)	41 (3.5)	0.005
QREC	50.7	1,332 (54.6)	699 (55.7)	633 (53.5)	0.276
CtxCroR-kpn	31.1	762 (44.4)	379 (42.6)	383 (46.3)	0.144
CR-kpn	10.9	207 (12.1)	93 (10.5)	114 (13.8)	0.042
CR-pae	18.3	293 (22.1)	144 (21.0)	149 (23.3)	0.355
CR-aba	53.7	655 (56.2)	366 (57.6)	289 (54.4)	0.298
Total	–	3,912 (38.6)	2,057 (39.0)	1,855 (38.1)	0.366

*CARSS, China Antimicrobial Resistance Surveillance System.

**Figure 1:**
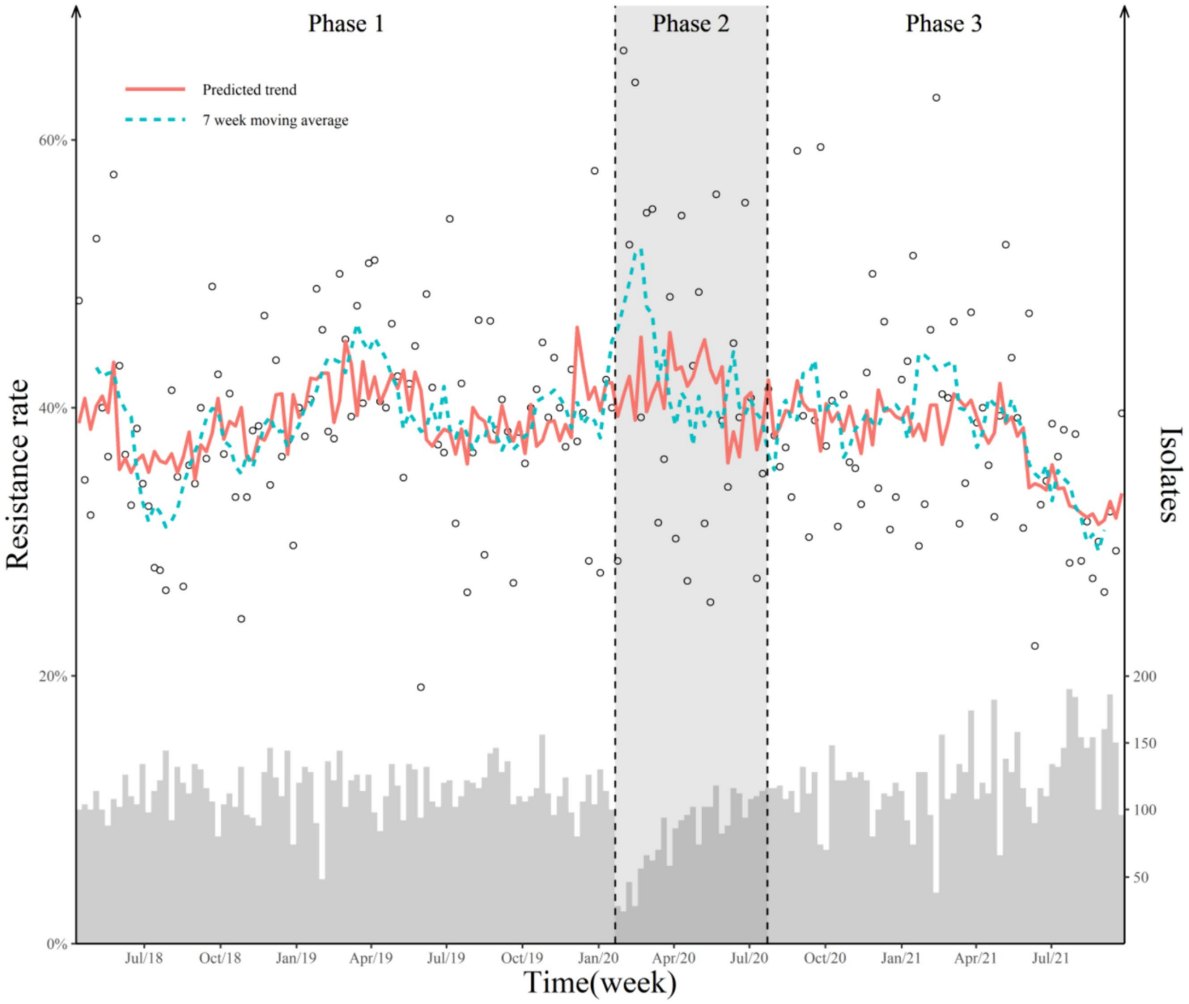
Weekly antimicrobial-resistant rate and predicted trend with two breakpoints. The hollow dot represents the weekly point prevalence of antimicrobial-resistant bacteria. The histogram represents the number of isolates detected. Two dashed lines represent the COVID-19 outbreak and the official release of AMS policy. The red solid line represents the predicted value of the Poisson segmented regression model. The blue dotted line represents the 7-week moving average of the antimicrobial-resistant rate. “Jul/18” in the X-axis means July 2018.

Taking the COVID-19 measures as the only interruption time point, we found a significant decline in the weekly prevalence of AMR following the COVID-19 outbreak and the launch of COVID-19 measures (Table 3). The two-point interruption time series analyses showed that the AMS policy was the only effective policy intervention, with a 0.42% (95% CI 0.05–0.80) WPC (Table 3; Figure 1).

**Table 3:** Weekly percentage change of the prevalence of antimicrobial resistance: results from interrupted time series analyses.

Interrupted time series modeling	Parameter evaluated^*^	Weekly percentage change (%)	*p*	95%CI	
**One interruption point only**					
COVID-19	Baseline trend	0.04	0.680	−0.15	0.23
	Step change	7.15	0.329	−6.74	23.07
	Trend change	−0.29	0.044	−0.57	−0.01
IPC policy	Baseline trend	0.08	0.377	−0.10	0.25
	Step change	0.38	0.957	−12.67	15.37
	Trend change	−0.29	0.044	−0.58	−0.01
Second IPC policy	Baseline trend	0.06	0.430	−0.08	0.19
	Step change	1.52	0.828	−11.48	16.37
	Trend change	−0.36	0.029	−0.69	−0.04
AMS policy	Baseline trend	0.03	0.619	−0.10	0.17
	Step change	5.20	0.471	−8.37	20.69
	Trend change	−0.41	0.017	−0.75	−0.07
**Two interruption points**					
COVID-19 + IPC policy	Baseline trend	0.03	0.743	−0.16	0.22
	COVID step change	15.86	0.243	−9.99	47.66
	Policy step change	−11.25	0.345	−30.41	14.3
	Policy trend change	−0.24	0.110	−0.54	0.06
COVID-19 + Second IPC policy	Baseline trend	0.05	0.636	−0.14	0.24
	COVID step change	1.16	0.891	−14.2	19.19
	Policy step change	0.99	0.901	−13.56	18.07
	Policy trend change	−0.35	0.058	−0.71	0.01
COVID-19 + AMS policy	Baseline trend	0.04	0.650	−0.15	0.24
	COVID step change	−1.21	0.881	−15.75	15.8
	Policy step change	5.68	0.471	−9.07	22.85
	Policy trend change	−0.42	**0.027**	−0.80	−0.05

*Parameters adjusted for variations in season, gender, age, service setting, care unit, primary site of infection and Gram type of bacteria were fitted as covariates. IPC, infection prevention and control practices; AMS, antimicrobial stewardship.

**Figure 2:**
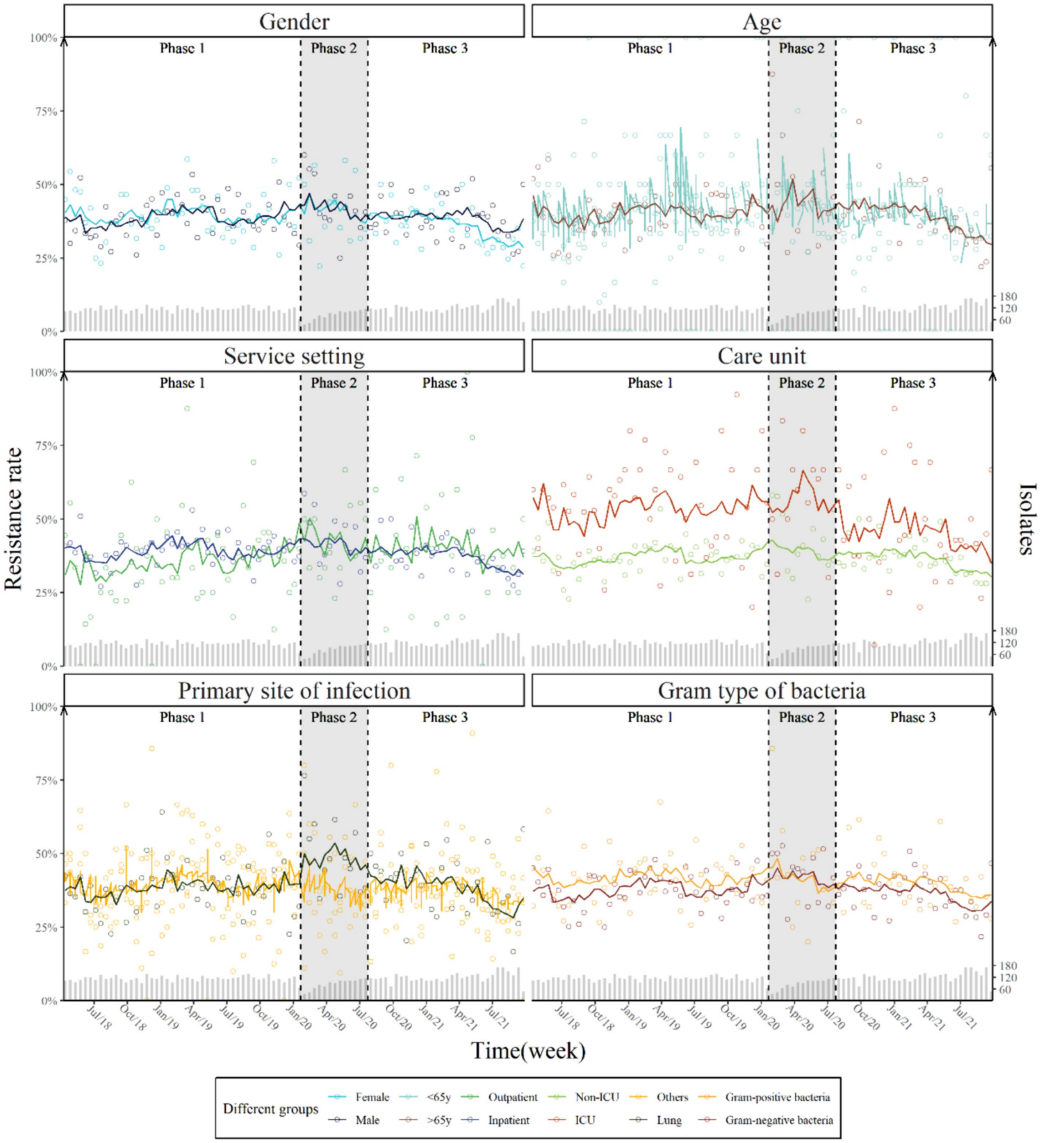
Weekly prevalence and predicted trend of antimicrobial resistance by gender, age, service setting, care unit, primary site of infection and Gram type of bacteria with two breakpoints.

The subgroup analyses found no significant differences in step or trend changes by gender, age, service setting, care unit, sample origin, and bacteria type, although isolates taken from the ICU had consistently higher prevalence of AMR (IRR = 1.34, 95% CI [1.03–1.73]) (Supplementary Table S3). No significant changes in the weekly prevalence of AMR were found following the launch of COVID-19 measures (Figure 2).

## Discussion

Our study found a significant long-term decline in weekly antimicrobial-resistant rates following the COVID-19 pandemic, particularly following the adoption of the management policy on rational use of antimicrobial agents. Given a low-level COVID-19 patient population (25), the problem of COVID-19 patients’ antibiotic abuse in China was not as serious as in other countries (26), which would enhance the management of rational use of antimicrobial agents in reducing the spread of antimicrobial resistance (27, 28). A worldwide systematic review shows that The COVID-19 pandemic may have hastened the emergence and transmission of AMR, particularly for Gram-negative organisms in hospital settings, furthermore, the absence of IPC and/or AMS initiatives was associated with an increase in gram-negative AMR (risk ratio 1.11, 95% CI: 1.03–1.20), which was consist with our results. The hospital, which serves as both a large tertiary referral center and a designated facility for treating local COVID-19 patients during the pandemic, has been affected by several factors that may have impacted the detection numbers of pathogens and the resistance rate. The early phase of the pandemic saw changes in healthcare-seeking behavior and reduced access to healthcare, potentially leading to undiagnosed and untreated bacterial infections (29). There is increasing evidence that the effects of COVID-19 on MDROs vary, and specific antimicrobial stewardship measures should be prioritized over existing empiric treatment guidelines (30).

Prior research has indicated that the transfer of Multi-Drug Resistant Organisms (MDROs) was detected in 18.5% of patients, with this transfer often occurring shortly after admission (31). In regions heavily impacted by the COVID-19 crisis, the combination of a higher patient density and greater severity of illness, along with an increased workload and reduced space, may facilitate the transmission of drug-resistant bacteria between healthcare workers and patients (32).

Compared with other departments, we found the baseline rate of antimicrobial resistance in ICU was significantly higher. Indeed, multidrug-resistant bacteria infections are more likely to occur in patients with advanced co-morbid illnesses, prolonged hospital stays, use of invasive procedures, and prior antibiotic exposure, which are more common in the ICU (33). Besides, we did not see any discernible variations in step or trend changes of antimicrobial resistance between different genders (male vs. female), ages (<65 vs. ≥65 years), service settings (outpatient vs. inpatient), care unit (ICU vs. non-ICU), the primary site of infection (Lung vs. others), or Gram type of bacteria (positive vs. negative). The results of the two large sample systematic reviews are not the same, which may be due to the individualized differences of the included populations. A meta-analysis showed that the prevalence of secondary bacterial infection was higher in ICU patients than in non-ICU patients. Another meta-regression showed that patient age and comorbidities (such as diabetes, hypertension, COPD, etc.) significantly affected the resistance prevalence of COVID-19 patients. The management of rational use of antimicrobial agents was likely to be efficient in most cases since China had not experienced a surge of COVID cases. However, things have changed after abandoning the zero-COVID policy, and the policy intervention may not work in the context of high numbers of COVID-19 cases (34). Under this context, regular surveillance and precise evaluation would become even more crucial (35).

After the COVID-19 outbreak, we found significantly increasing antimicrobial-resistant rates in PRSP, CR-eco and CR-kpn. Although the sample size of these strains is too small to have a significant impact on the overall AMR trend, the increase in AMR relating to the three strains has important clinical implications. For instance, *S. pneumoniae* is susceptible to penicillin and is a major cause of community-acquired pneumonia, meningitis, sepsis, bacteremia and otitis media, whose treatment would become problematic due to the increasing antimicrobial resistance (36). Similarly, CR-eco and CR-kpn usually cause a wide range of serious infections that carry significant morbidity and mortality and infections lack new agents in clinical practice (37, 38). The lack of effective treatments for infections involving multidrug-resistant *Klebsiella pneumoniae* infections may result in higher risks of mortality and disease burden, which merits attention. Our results showed that after the outbreak of the pandemic, the risk of CR-kpn decreased significantly, which was consistent with recent research (39, 40). There were also increasing cases of carbapenems-resistant *Enterobacter* after the COVID-19 outbreak (41–44). However, the study based on the national AMR surveillance data in Japan discovered a drop in patients with PRSP from 2019 to 2020 (45). The different trend in China may be explained by hospitals and doctors’ strong preference for penicillin and cephalosporin whose proportion of prescriptions was still increasing in 2020 (16).

Since China relaxed its zero-COVID policy, the demand for nucleic acid detection has been sharply falling, allowing many laboratories to focus on the detection of dangerous bacteria and antimicrobial resistance (46). Focus should be placed on developing diagnostic tests that distinguish between bacterial infections and COVID-19, particularly multiplex diagnostic tests that target viruses and bacterial infections, to reduce the need for unnecessary antimicrobials (47).

There are several limitations in this study. First, even though antimicrobial-resistant rates in our study were close to the data reported by CARSS, our study was conducted in a single hospital which was not representative of some hospitals in different settings. Second, our study was unable to explain why antimicrobial-resistant rates had changed due to the lack of antimicrobial prescription data, which reduces the strength of our conclusions. Finally, we also did not consider the lag effects of interventions. Further multicenter studies are expected to be conducted.

## Conclusion

During the study period, the overall status of antimicrobial resistance in teaching hospital has improved as a result of COVID-19’s effective implementation of antimicrobial stewardship, but there is still the problem of the increase in the resistance rate of some important clinical pathogens. Regular monitoring and adjustment of prevention and control strategies are very essential to respond to the major change in the epidemic prevention and control policy.

## Data availability statement

The original contributions presented in the study are included in the article/supplementary material, further inquiries can be directed to the corresponding authors.

## Ethics statement

Ethics approval was obtained from the Ethics Review Committee of the School of Public Health, Sun Yat-sen University (No. 2021-117), and the requirement for written informed consent from patients was waived.

## Author contributions

WL: Conceptualization, Data curation, Formal analysis, Methodology, Software, Validation, Writing – original draft, Writing – review & editing. XY: Conceptualization, Data curation, Formal analysis, Methodology, Software, Writing – original draft, Writing – review & editing. CL: Conceptualization, Data curation, Methodology, Resources, Writing – review & editing. XuL: Conceptualization, Formal analysis, Resources, Writing – review & editing. LS: Conceptualization, Data curation, Formal analysis, Writing – review & editing. YZ: Conceptualization, Data curation, Formal analysis, Writing – review & editing. HX: Conceptualization, Data curation, Formal analysis, Writing – review & editing. JL: Conceptualization, Formal analysis, Resources, Writing – review & editing. MZ: Conceptualization, Formal analysis, Resources, Writing – review & editing. SY: Conceptualization, Formal analysis, Resources, Writing – review & editing. XiL: Conceptualization, Formal analysis, Resources, Writing – review & editing. BH: Conceptualization, Resources, Writing – review & editing, Formal analysis. LY: Conceptualization, Data curation, Formal analysis, Funding acquisition, Investigation, Methodology, Project administration, Resources, Supervision, Validation, Writing – original draft, Writing – review & editing.
